# Synthesizing existing evidence to design future trials: survey of methodologists from European institutions

**DOI:** 10.1186/s13063-019-3449-6

**Published:** 2019-06-07

**Authors:** Adriani Nikolakopoulou, Sven Trelle, Alex J. Sutton, Matthias Egger, Georgia Salanti

**Affiliations:** 1 Institute of Social and Preventive Medicine (ISPM), University of Bern, Bern, Switzerland; 2 CTU Bern, University of Bern, Bern, Switzerland; 3 Department of Health Sciences, College of Medicine, Biological Sciences and Psychology, University of Leicester, Leicester, UK

**Keywords:** Conditional trial design, Sample size, Meta-analysis, Network of interventions

## Abstract

**Background:**

‘Conditional trial design’ is a framework for efficiently planning new clinical trials based on a network of relevant existing trials. The framework considers whether new trials are required and how the existing evidence can be used to answer the research question and plan future research. The potential of this approach has not been fully realized.

**Methods:**

We conducted an online survey among trial statisticians, methodologists, and users of evidence synthesis research using referral sampling to capture opinions about the conditional trial design framework and current practices among clinical researchers. The questions included in the survey were related to the decision of whether a meta-analysis answers the research question, the optimal way to synthesize available evidence, which relates to the acceptability of network meta-analysis, and the use of evidence synthesis in the planning of new studies.

**Results:**

In total, 76 researchers completed the survey. Two out of three survey participants (65%) were willing to possibly or definitely consider using evidence synthesis to design a future clinical trial and around half of the participants would give priority to such a trial design. The median rating of the frequency of using such a trial design was 0.41 on a scale from 0 (never) to 1 (always). Major barriers to adopting conditional trial design include the current regulatory paradigm and the policies of funding agencies and sponsors.

**Conclusions:**

Participants reported moderate interest in using evidence synthesis methods in the design of future trials. They indicated that a major paradigm shift is required before the use of network meta-analysis is regularly employed in the design of trials.

**Electronic supplementary material:**

The online version of this article (10.1186/s13063-019-3449-6) contains supplementary material, which is available to authorized users.

## Introduction

Systematic reviews can identify knowledge gaps that may direct the research agenda toward questions that need further investigation. Knowledge gaps may arise when the available data are insufficient, or when there is no evidence at all that can answer a research question. Once identified, primary research (e.g., trials) may be designed and conducted to fill such gaps.

Such considerations, along with implementation strategies, have appeared in the literature. The Agency of Healthcare Research and Quality developed a framework for determining research gaps using systematic reviews [1]. Methods for informing aspects of trial design based on a pairwise meta-analysis have also been proposed and include powering a future trial based on a relevant existing meta-analysis [2–4] or investigating how a future trial would alter the meta-analytic summary effect obtained thus far [5, 6]. These methods are limited to situations in which existing evidence consists of two interventions. When existing evidence forms a network of interventions, synthesis of available trials can be done using network meta-analysis. Network meta-analysis is increasingly used in health technology assessment (HTA) to summarize evidence and inform guidelines [7]. However, its potential to inform trial design has not received much attention.

Methodological developments that use network meta-analysis as a basis for further research [3, 8] have been recently collated to form a holistic framework for planning future trials based on a network of interventions [9]. The framework, called ‘conditional trial design’, combines considerations relevant to both evidence synthesis and trial design; ‘conditional’ refers to the fact that the design of a new study depends (is conditional) on the existing evidence. The framework consists of three parts. The first part asks whether the existing evidence answers the research question*.* This part pertains to interpreting meta-analysis results, which is related to deciding whether existing evidence is conclusive, whether multiple testing is needed when a meta-analysis is regularly updated, and how to interpret evidence from multiple outcomes. The second part of the framework is related to how best to use the existing evidence to answer the research question. The third and last part of the framework addresses how to use the existing evidence to plan future research. The conditional trial design requires that the assumptions of network meta-analysis are plausible and that the credibility of the results is high. In the case of violation of the transitivity assumption (that for each comparison there is an underlying true relative treatment effect which applies to all studies regardless of the treatments compared), or in the presence of studies with a high risk of bias, the existing network of interventions would not provide reliable evidence and thus should not be used to inform the planning of new studies.

We conducted a survey of views on the feasibility of the conditional trial design among trial statisticians, methodologists (researchers developing methodology), and users of evidence synthesis research. To this aim, the survey included questions relevant to the three parts of the conditional trial design. In particular, our objectives were to capture opinions and current practices regarding: 1) the decision about whether a meta-analysis answers the research question (first part); 2) the acceptability of network meta-analysis as a technique to enhance the evidence and answer the research question (second part); and 3) the use of evidence synthesis in the planning of future clinical research (third part).

## Methods

### Invited participants

Our convenience sample consisted of researchers working in Europe either in nonprofit organizations or in the pharmaceutical industry. We contacted researchers from the World Health Organization (WHO), 13 HTA agencies, 17 pharmaceutical companies or companies that prepare HTA submissions, and all clinical trial units in the UK, Norway, Switzerland, and Germany. The full list of contacted organizations can be found in Additional file 1. We sent a brief description and the link to the survey by email to key personnel within each organization, which included a request to forward it to anyone within their organization who might be interested, or we sent email messages to a mailing list or individuals. We did not track whether an invited person completed the survey, and we sent no reminders.

### Survey design

**Fig. 1:**
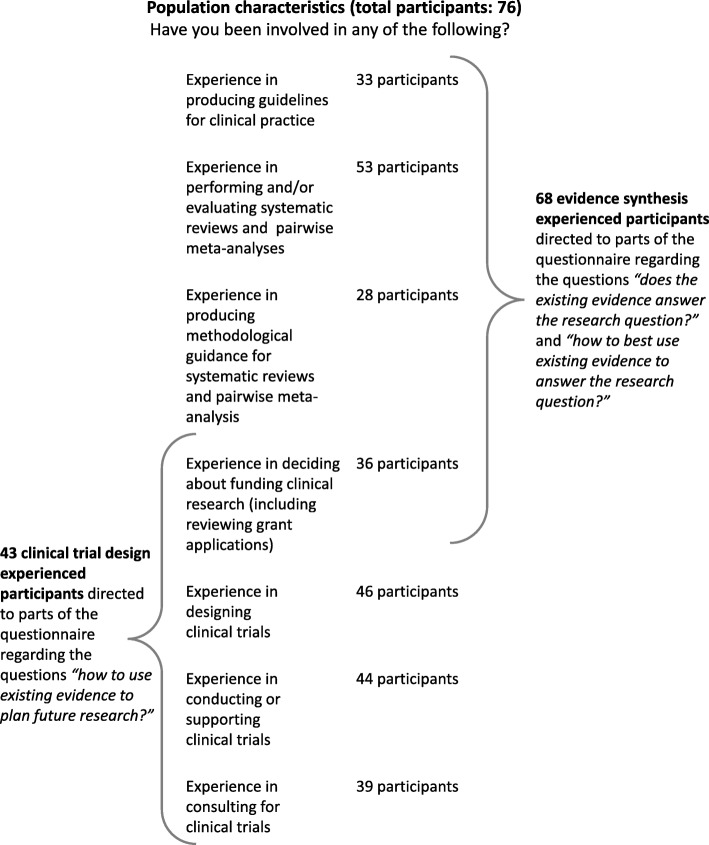
Schematic representation of the parts of the survey to which participants were directed according to their involvement in several aspects of systematic reviews, guidelines, and clinical trials production

We designed an online questionnaire of 24 questions which would take around 15–20 min to complete using Survey Monkey (http://www.surveymonkey.com). We started with questions regarding principal affiliation, experience with systematic reviews, meta-analysis, network meta-analysis, guidelines, clinical trials, and involvement in research funding decisions. Implementation of the framework on which we wanted to capture opinion would require a collaborative process between experienced researchers in the areas of evidence synthesis and trial design. Participants were therefore directed to one or both of the survey’s main parts, depending on their expertise, as shown schematically in Fig. 1. For the majority of the questions, it was possible to select more than one answer. The full questionnaire is presented as Additional file 2. The survey was open between 10 October 2016 and 9 December 2016. Responses were collected anonymously. A pilot version of the questionnaire was tested with three statisticians and two methodologists from the Clinical Trials Unit and Institute of Social and Preventive Medicine of the University of Bern.

The first part of the survey concerned current practices in deciding whether a meta-analysis answers the research question at hand. Only participants experienced in evidence synthesis and those who had been involved in deciding about funding clinical research were directed to this part. Certain questions asked participants to choose or report what they are actually doing*,* in practice, while others asked participants to choose what they think should be done. Topics related to interpretation of the meta-analysis results, how multiple outcomes are integrated, and issues of multiple testing in the context of a continuously updated meta-analysis. A separate section covered issues related to the acceptability of network meta-analysis.

The next part of the survey contained questions about the use of evidence synthesis, as pairwise or network meta-analysis, for the design of clinical trials. For all questions in this part, the term clinical trials referred to randomized, post-marketing (e.g., phase IV) controlled clinical trials. Participants experienced in clinical trials and those who declared involvement in funding decisions were directed to this part (Fig. 1). Some of the questions were formulated so that the participants answered them in their capacity as citizens who fund research (such as EU-funded clinical trials or other research funded by national funds through their taxation).

### Analysis

We derived descriptive statistics as frequencies and percentages for participants’ characteristics (affiliation, job role, experience in meta-analysis and clinical trials). Percentages include missing responses in the denominator. Some questions allowed or requested free text answers by participants; we present some illustrative written quotes regarding participants’ willingness to consider a clinical trial design informed by meta-analysis and the biggest barriers to adopting such a design. Where a visual analogue scale was used and for the question of rating clinical research proposals submitted for funding, median, 25th, and 75th percentiles are presented. As a post-hoc analysis, we used a Pearson’s Chi-squared test to examine whether level of experience with evidence synthesis and clinical trials was related to different views on the acceptability of network meta-analysis and participants’ likelihood to consider the use of conditional trial design. Whenever any expected frequency is less than 1 or at least 20% of cells had expected counts of 5 or less, a Fisher’s exact test was used instead of a Pearson’s Chi-squared test. The rest of the analyses were planned prospectively. All analyses were performed using Stata 14.1.

## Results

### Participants characteristics

In total, 76 researchers completed the survey, of whom 29 (38%) were affiliated with a clinical trial unit and 15 (20%) with the pharmaceutical industry. Fifty-three participants (70%) had performed and/or evaluated a systematic review, 46 (61%) had designed a clinical trial, and 36 participants (47%) had been involved in decisions about funding clinical research including reviewing grant applications.

The involvement of researchers in trials, meta-analyses, and network meta-analyses varied. Sixty-three researchers (83%) had been involved in at least one clinical trial, over half of whom (33) had been involved in more than 20 trials. Sixty-one researchers (80%) reported involvement in at least one pairwise meta-analysis, while 34 (45%) had participated in one or more network meta-analyses. The complete characteristics of participants can be found in Table 1.

**Table 1:** Opinions and practices of participants regarding evidence-based planning of future trials

Question	Possible answers	Responses (%)
What is your primary affiliation?	Clinical trials unit	29 (38%)
	A funding body	3 (4%)
	Pharmaceutical industry	15 (20%)
	HTA/Cochrane/WHO	28 (37%)
	Missing	1 (1%)
How do you judge whether a summary treatment effect provides conclusive evidence or whether further research is needed (more than one choice allowed)?	I examine the statistical significance of the summary effect and its CI	31 (46%)
	I examine the clinical importance of the summary effect and its CI	39 (57%)
	I test whether future studies could change the statistical significance of the summary effect	7 (10%)
	I follow the GRADE guidelines for judging imprecision	19 (28%)
	Not involved in interpretation of meta-analysis results/other/missing	29 (43%)
Do you think that network meta-analysis should be considered as the preferred evidence synthesis method instead of pairwise meta-analysis?	Yes, network meta-analysis should always be preferred	15 (22%)
	No, network meta-analysis should not be considered	5 (7%)
	It should be considered only if there are no or few direct studies	25 (37%)
	Other/missing	23 (34%)
According to your experience, results from relevant meta-analyses are considered to (more than one choice allowed):	Define the alternative effect size in power calculations	25 (58%)
	Decide about the intervention in the comparator arm	19 (44%)
	Define other parameters involved in sample size calculations	29 (67%)
	Define health outcomes to be monitored	22 (51%)
	Other/missing	7 (16%)
What do you think is the biggest barrier towards adopting the conditional trial design in designing trials?	Lack of training	6 (14%)
	Changing the paradigm of funders and researchers	16 (37%)
	Lack of good-quality meta-analyses	4 (9%)
	Other/missing	17 (40%)
Question	Research proposals	Median (25th to 75th percentile)
As a citizen supporting publicly funded research how would you rank (from 1 being the top priority to 5 being the least) the following proposals tackling the treatments for an important health condition? Consider also the cost for each research proposal (presented in parenthesis in arbitrary units).	A well-powered three-arm randomized trial comparing the three most promising interventions (none of which is standard care) (100)	4.0 (3.0 to 5.0)
	A well-powered three-arm randomized trial comparing the two most promising interventions and standard treatment (90)	2.0 (1.0 to 2.0)
	A well-powered two-arm randomized trial comparing a newly launched treatment and standard treatment (70)	3.0 (2.0 to 4.0)
	A large registry involving many countries (40)	5.0 (3.5 to 5.0)
	A network meta-analysis comparing all available treatments using existing studies (10)	1.5 (1.0 to 3.0)

The full text and questions are presented in Additional file 2

*CI* confidence interval, *GRADE* Grading of Recommendations Assessment, Development and Evaluation, *HTA* health technology assessment, *WHO* World Health Organization

### Does the existing evidence answer the research question?

Among the 76 participants, 68 (89%) had experience in evidence synthesis and answered questions related to the first part of the conditional trial design framework which is relevant to the interpretation of meta-analysis results (Fig. 1).

When asked about judging when a summary treatment effect is conclusive and when further research is needed, 39 of these 68 researchers (57%) examined the clinical importance of the summary effect, while slightly fewer (31) examined the statistical significance of the summary effect (Table 1). Most participants examining the statistical significance of the summary effect also examine its clinical importance (28 participants, 37%).

Participants were asked about adjustment for multiple testing issues when a meta-analysis is updated with new studies. Twenty-two of the 68 participants (32%) indicated that adjustment for multiple testing is not required for a repeatedly updated meta-analysis, while 18 participants (27%) reported that such an adjustment is required. The rest (28 participants, 41%) either did not respond or indicated that they did not know. Participants were also asked about interpreting evidence from multiple outcomes that bears upon a preference for one of two treatments. Among the 68 participants, 25 (37%) reported involving stakeholders in deciding which outcomes are more important, while 22 participants (32%) used methods described in the recommendations of the Grading of Recommendations Assessment, Development and Evaluation (GRADE) working group.

### How best to use the existing evidence to answer the research question?

The 68 participants who had experience in evidence synthesis were directed to answer questions regarding the second part of the conditional trial design: how to use the existing evidence to answer the research question (Fig. 1).

Asked whether they prefer network meta-analysis as an evidence synthesis method to pairwise meta-analysis, participants indicated a comparatively low preference for network meta-analysis. Among the 68 participants, 15 (22%) preferred network to pairwise meta-analysis. A total of 25 participants (37%) indicated that network meta-analysis should be considered when there are either no or very few direct studies (Table 1). Eight participants suggested other approaches as indicated by two of their responses: “I would look at both direct and indirect analysis” and “I see the evaluation as one process and don’t want to disregard one versus the other”.

**Fig. 2:**
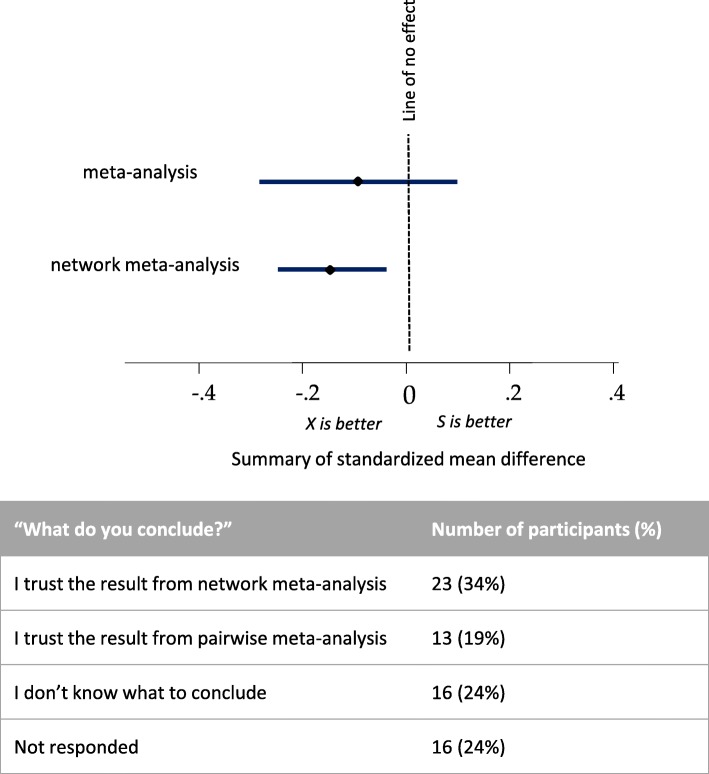
Opinions among researchers on their interpretation of a hypothetical scenario where network meta-analysis provides conclusive evidence that treatment X is better than treatment S while pairwise meta-analysis indicates that further evidence is needed. The question was addressed to the subset of 68 ‘evidence synthesis-experienced’ participants

When asking participants about their interpretation in a more specific scenario, such as the one presented in Fig. 2, nearly twice as many participants indicated that they trusted network meta-analysis more than pairwise meta-analysis when the results are more precise (23 versus 13 participants). A considerable subgroup of participants claimed that they did not know what to conclude, or they did not respond to the question (32 total participants, 48%) (Fig. 2).

### How to use the existing evidence to plan future research?

Among the total of 76 participants, 43 researchers experienced in clinical trial design (57%) were directed to questions related to the third part of the conditional trial design, which is relevant to practices and opinions about using meta-analysis to inform aspects of the design of future clinical trials (Fig. 1).

#### Practices of using meta-analysis in the design of clinical trials

Participants rated their use of evidence synthesis in the design of clinical trials on a visual rating scale from 0 (never) to 1 (always). The median value was 0.44 (25th percentile 0.22, 75th percentile 0.67). A total of 29 participants (67%) reported using meta-analyses of previous trials in the determination of other parameters involved in sample size calculations (such as standard deviations, baseline risk, and so on), 25 participants (58%) considered meta-analyses in defining alternative effect sizes in power calculations, and 22 (51%) used meta-analyses in the determination of health outcomes to be monitored (Table 1).

When asked about the best among five approaches to resolve uncertainty regarding the best pharmaceutical treatment for a given condition, a three-arm randomized trial comparing the two most promising interventions and standard treatment, and a network meta-analysis comparing all treatment alternatives were the most popular options (rating medians 2.0 and 1.5, respectively). The least favorable research design was a large international registry (rating median 5.0, Table 1). The rating frequencies for each research proposal are given in Additional file 3.

#### Acceptability of sample size calculations based on an existing meta-analysis

Twenty-six participants (60%) were aware of the methodology of explicitly incorporating results from a meta-analysis in the sample size calculation of a future trial (based on conditional power). Ten participants (23%) said they would consider the approach when planning a trial in the future and another 18 (42%) responded that they would possibly consider it. Half (22 participants, 51%) were aware of the methodology and indicated that they were willing to consider it. When asked about reasons for not considering such a design, participants justified their answers with arguments mainly associated with concerns about the reliability and validity of the meta-analysis as well as the paradigm of perceiving trials as independent pieces of evidence. Some sample answers are presented in Table 2. When asked to respond from the perspective as citizens supporting publicly funded research, 21 of the 43 participants (49%) indicated that priority should be given to conditional trial design compared with conventional sample size calculations. Changing the paradigm that trials should be independent experiments was presented as the biggest barrier towards adopting such a trial design (16 participants, 37%) (Table 1).

**Table 2:** Key free text quotes from responses

**From respondents who answered “No” or “Possibly” to the question “Would you be willing to consider a conditional trial design next time you plan a trial?”**	
• “Lots of examples where a large definitive trial has contradicted the results of a meta-analysis of smaller trials”	
• “Any meta-analysis is observational research”	
• “Because when you finalize the trial, the meta-analysis will be outdated. Your study should be a standalone trial”	
• “Not enough faith in the homogeneity/comparability of the studies”	
• “The assumptions behind a meta-analysis (homogeneity, no publication bias), are very rarely plausible, so a typical RCT has to offer a chance of providing a definitive conclusion on its own”	
• “Clinical trials are perceived as independent pieces of evidence. There would need to be a major shift by regulators, HTA bodies and physicians for companies to design trials in the context of meta-analyses”	
• “Usually the context in which I work is of trials supporting applications for a license. Regulators require each study to be ‘significant’ independently of others”	
• “Wonder whether it would be convincing to authorities”	
• “In the regulatory context, meta-analyses are typically NOT considered for approval decisions, at least not directly. (Typically). I would answer differently for publicly funded studies. A newish suggestion—most of our trials are phase II/III, where things are a little different”	
**From respondents who replied “Other” to the question “What do you think is the biggest barrier towards adopting conditional trial design in designing trials?”**	
• “Although trials can be planned to add just enough power to an existing meta-analysis, there is a high risk that such planning fails because of wrong assumptions, differences in study execution, or other reasons”	
• “It is flawed and too risky (why give an experimental drug in an underpowered study)”	
• “Guidelines from important regulatory and health economic agencies”	
• “Lack of dissemination”	
• “Skepticism as trials should be powered to stand alone, I would think. All other studies in the MA may not be comparable or of high quality”	
• “It’s not necessarily logical”	
• “I don’t believe this is an appropriate way to design trials”	

*HTA* health technology assessment, *MA* meta-analysis, *RCT* randomized controlled trial

### Relation between level of experience with clinical trials/evidence synthesis and acceptability of network meta-analysis and conditional trial design

Experienced researchers in evidence synthesis were more likely to have confidence in network meta-analysis. Among the 27 participants with experience in evidence synthesis who indicated that they either can perform network meta-analysis themselves or have been involved in systematic reviews with network meta-analysis, 11 (41%) responded that, in general, network meta-analysis is preferable to pairwise meta-analysis. Among the 41 participants with little or no experience with network meta-analysis, only four (10%) said that network meta-analysis is to be preferred (Pearson’s Chi-squared test *P* value 0.003, Additional file 3).

The willingness to consider the use of an existing meta-analysis to inform sample size calculations of a new study did not materially vary according to researchers’ experience in clinical trials or evidence synthesis (Additional file 3).

## Discussion

In this survey of methodologists based in Europe, participants reported low to moderate use of evidence synthesis methods in the design of future trials. Evidence synthesis is used for the design of around half of the trials. The information most used relates to the parameters required for sample size calculations and outcome definitions. Our results broadly agree with those of Clayton et al. who found that 50% of investigators who responded to their survey had used meta-analysis to inform a future trial [10]. The scope of the survey by Clayton et al. was similar to ours but it did not focus on issues pertaining to interpreting evidence synthesis and acceptability of network meta-analysis.

Empirical evidence has shown lower uptake of systematic reviews in planning new trials than the findings in the current survey and the survey by Clayton et al. [11–19]. Clarke et al. assessed reports of randomized trials published in *Annals of Internal Medicine*, *BMJ*, *JAMA*, *The Lancet*, and the *New England Journal of Medicine* in the month of May in the years 1997, 2001, 2005, and 2009. According to their findings, only a small proportion of trial reports attempted to integrate their findings with existing evidence [11, 12, 15, 16]. Out of 446 trial protocols submitted to the UK ethics committees in 2009, only four (less than 1%) used a meta-analysis and 92 (21%) used previous studies to define the treatment difference sought [20]. A review of 1523 trials published from 1963 to 2004 showed that fewer than 25% of relevant previous randomized controlled trials were cited by subsequent randomized controlled trials [21].

Funders of clinical trials often emphasize the importance of using existing evidence in grant applications [14, 22, 23]. Thirty-seven (77%) out of 48 trials funded by the National Institute for Health Research (NIHR) Health Technology Assessment program between 2006 and 2008 referenced a systematic review in the funding application; the percentage was 100% for trials funded in 2013 [24]. The interest of funders in research synthesis dates back to the 1990s when several organizations responsible for funding clinical research started to require systematic reviews of existing research as a prerequisite for considering funding for new trials [14]. But as Clayton et al. point out, it is not clear to what extent and in which way funders expect evidence synthesis to be used [10]. Nasser et al. searched the websites of 11 research funding organizations and, while four of them require systematic reviews to show that new clinical trials are needed, only the NIHR requires reference to relevant systematic reviews [22]. We did not specifically survey bodies that fund clinical trials (such as the NIHR or the Swiss National Science Foundation). A survey of funding agencies along with a review of their guidance on how trialists should use existing evidence when designing and implementing new trials would be an important step forward.

Our study has some limitations that render the generalizability of its results questionable. First, the sample size of our survey was 76 participants, which is relatively small; a bigger sample size would allow us to produce more precise estimates for the outcomes of interest. Furthermore, using referral or snowball sampling means that we could not estimate the response rate for our survey. Second, we cannot exclude the possibility that the characteristics of participants systematically differed from those who either did not receive the questionnaire or received it but decided not to participate. Such nonresponse selection bias seems likely considering that a relatively high proportion of participants knew about calculating sample size based on a meta-analysis (60%), despite the fact that the methods have only recently been developed [2, 8, 9] and, in our experience, are not widely used. This indicates that the participants were probably a well-informed sample of methodologists who were up to date with recent developments. Moreover, the questionnaire has not been independently validated and some terms used might have different meaning for researchers with different backgrounds. A follow-up survey on a larger scale, including representatives from funding agencies, could provide more information on the potential of using existing evidence in the design of new studies.

We clarified in the survey that the term “clinical trials” should mean “randomized, post-marketing (e.g., phase IV) controlled clinical trials”. This clarification was made because usually little evidence is available before licensing which constitutes an important barrier to using the proposed method. However, it might be that trials examining licensed treatments are considered phase III because of their size and scope. Clearer guidance on how comparative effectiveness data can and should be used in the entire process of approval and adoption of new drugs would be of interest [25, 26].

This survey indicates a lack of consensus in aspects related to the interpretation of meta-analysis results. None of the answers to the question regarding interpreting evidence from multiple outcomes was selected by more than about a third of participants. Participants also did not agree on the use of adjustment for multiple testing when a meta-analysis is updated. This lack of consensus is in line with the lack of agreement about using sequential methods in the literature. Opinions range from regularly using sequential meta-analysis [27, 28], to adjusting for repeated updates in specific cases [29–31], to never correcting summary treatment effects using sequential methods [32]. Concerns about the reliability of meta-analysis affect the acceptability of the conditional trial design; we think, however, that such concerns are likely to diminish over time as meta-analysis is increasingly used for decision-making and guideline development. The second main pillar of skepticism towards the conditional trial design is the perception of trials as independent experiments. It will be interesting to see whether this view will be challenged in the light of increasing awareness of research waste.

Resources for health research are limited and thus an economical and ethical allocation of funds for clinical trials requires minimizing human and monetary costs and risks. While certain research funders, clinical trial planners, and journal editors acknowledge the need to consult the existing evidence base before conducting a new trial, in practice these considerations are not concrete and explicit and quantitative methods are rarely used. We propose that clinical trialists explicitly report (e.g., in published protocols) how they will compute the sample size of their planned trials including the way in which they will use existing evidence, for example by defining the alternative effect size, the intervention group risk, or by computing the conditional power of the planned trial. Further research on ways in which evidence synthesis can be efficiently used in the planning of new trials could use, and possibly combine, considerations from value of information analysis, adaptive design methodology, and formal decision analytic methods. Funding agencies and journal editors could contribute to preventing waste by establishing concrete policies on the use of existing evidence when assessing requests for funding or publishing trials.

## Additional files

Additional file 1: List of invitations. (DOCX 22 kb)

Additional file 2: Questionnaire. (DOCX 82 kb)

Additional file 3: Full results. (DOCX 45 kb)

## Data Availability

The datasets used and/or analyzed during the current study are available from the corresponding author on reasonable request.
