# Supplementary material for: Synthesizing existing evidence to design future trials: survey of methodologists from European institutions
Source: Trials. 2019 Jun 7;20:334. doi: 10.1186/s13063-019-3449-6 (PMC6555919; doi:10.1186/s13063-019-3449-6)
Supplement: Supplementary file 1 — List of invitations. (DOCX 22 kb) [file 13063_2019_3449_MOESM1_ESM.docx]

Additional file 1

# List of invited entities

We emailed contact persons in WHO and in the following health technology assessment (HTA) agencies:

Zin (The Netherlands), IQWiG (Germany), KCE (Belgium), NICE (UK), AETSA (Spain), DIMDI (HTA based in Germany), LBI-HTA (Austria), HAS (France), NCPE (Ireland), NIPHNO (Norway), FINOHTA (Finland), SNHTA (Switzerland), Swedish Council of Technology Assessment interventions control (Sweden).

Key contact persons were invited from the following pharmaceutical companies:

*Amgen, Roche, Novartis, GlaxoSmithKline, AbbVie, Almirall, Merck, Ipsen, Eli Lilly, Actelion, Astra Zeneka, Griebsch Ingolf, Boehringer Ingelheim* and the companies *Mapi Values, Evidera* and *Amaris* that prepare HTA submission for some of the invited companies.

The clinical trials units (CTUs) in the UK were contacted via their co-ordinating centre in Leeds. In Norway CTUs were invited via their Norwegian correspondent for ECRIN (European Clinical Research Infrastructure Network).

Individual email messages were sent to all Swiss and German CTUs.
