# Supplementary material for: Synthesizing existing evidence to design future trials: survey of methodologists from European institutions
Source: Trials. 2019 Jun 7;20:334. doi: 10.1186/s13063-019-3449-6 (PMC6555919; doi:10.1186/s13063-019-3449-6)
Supplement: Supplementary file 2 — Questionnaire. (DOCX 82 kb) [file 13063_2019_3449_MOESM2_ESM.docx]

Additional file 2

Questionnaire to explore and evaluate the acceptability of evidence based planning of new research framework among different stakeholders

# General questions

## Choose your primary affiliation (Please choose only one. If you have many affiliations choose what relates to your primary occupation).

- Clinical Trials Unit
- A funding body (e.g. IMI, NIHR, SNF)
- Pharmaceutical industry
- A Health Technology Assessment organization
- The Cochrane Collaboration
- The World Health Organization

## Have you been involved in any of the following (all that apply)

1. Decide about funding clinical research (including reviewing grant applications)
2. Producing guidelines for clinical practice
3. Performing and/or evaluating systematic reviews and meta-analysis
4. Producing methodological guidance for systematic reviews and meta-analysis
5. Designing clinical trials
6. Conduct/support clinical trials
7. Consulting for clinical trials

If you chose either **a, b, c or d please complete Part I**

If you chose either **a, e, f or g please complete Part II**

## In how many trials have you been involved?

None, 1-5, 5-20, more than 20

## In how many meta-analyses have you been involved?

None, 1-5, 5-20, more than 20

## In how many network meta-analyses have you been involved?

None, 1-5, 5-20, more than 20, I don’t know what a network meta-analysis is

Part I

***Part I has 6 questions.***

***IMPORTANT INSTRUCTIONS***

- Some questions ask you to choose or report what **you do** in practice. *[please say what you do]*
- Some questions ask you to choose or report what you **believe/think** should be done in practice. *[please say what* ***you think should be done****]*

# Interpretation of meta-analysis results

*Consider many randomized clinical trials comparing an experimental intervention X to standard intervention S and their results are numerically synthesized using a meta-analysis aiming to provide evidence about clinical outcomes.* *When answering the following questions, assume that the studies have low risk of bias, low heterogeneity and the risk of publication bias is also low. The setting of the trials also enables you to generalize the meta-analysis findings when trying to answer a decision question.*

## Now consider only the numeric summary treatment effect from the meta-analysis for a single outcome. How **do you judge** whether it provides conclusive evidence or whether further research is needed? (tick any that apply). [please say what **you do** in practice]

## I examine the statistical significance of the summary effect and its confidence interval

## I examine the clinical importance of the summary effect and its confidence interval

- I test whether future studies could change the statistical significance of the summary effect
- I follow the GRADE guidelines for judging imprecision (considers the total sample size and the power of the analysis)
- I haven’t been involved in the interpretation of results from meta-analysis
- Other (specify)

## Assume now that you decided that the evidence is not conclusive and you want to repeat the meta-analysis every time that a new “X versus S study” is published until you have conclusive evidence. This arguably involves multiple testing because of the sequential nature of accumulating studies as they are published. Do you **think** that adjustment for multiple testing is required when you form your conclusions? [please say what **you think should be done**]

- Yes
- No
- I don’t know

## If your answer was yes, do you apply it in practice? (if your answer was “No”, or “I don’t know” skip the question) [please say what **you do** in practice]

- Yes
- No because I don’t know how
- No because it is not common practice in my organization

## To decide whether intervention X is preferable to intervention S you may want to consider the meta-analyses for several beneficial and harmful outcomes. How do you interpret evidence from multiple outcomes in forming your decision about the relative preference of X versus S? (tick any that apply). [please say what **you do** in practice]

- We use some formal decision analysis approach to weight the importance of beneficial and harmful outcomes (such as the ‘benefit-harm trade-off method’ or Multiple Criteria Decision Analysis, etc.)
- We use methods described in the ‘GRADE for recommendations’ approach to select a set of critical outcomes and then we summarize the results qualitatively
- We involve stakeholders to decide which outcomes are more important than others
- We don’t integrate results from different outcomes into a single conclusion
- We summarize results qualitatively in an informal way
- I haven’t been involved in the interpretation of results from a meta-analysis
- Other (specify)

# Acceptability of network meta-analysis

*Consider that there are many randomized trials comparing pairs of many competing interventions X, Y, Z … available for the same condition. These studies could be summarized to obtain the relative effect of any intervention compared to any other using network meta-analysis (NMA).*

## What is your level of experience with NMA (choose only one answer)?

- I can perform NMA
- I have been involved in systematic reviews with NMA but never done the statistical synthesis
- I have read systematic reviews with NMA
- I have heard about NMA but I don’t know much about it
- I don’t know what a NMA is

*The advantage of NMA is that it provides effect sizes for treatments that haven’t been compared in in any trial and also provides more precise estimates than those obtained by simple pairwise meta-analysis. However, NMA uses indirect evidence as well as direct evidence and hence rests on the assumption of consistency (that is, direct and indirect effects are in agreement).*

*In the following questions assume that the condition of consistency is deemed plausible (although inconsistency can never be excluded), heterogeneity is low and the risk of bias in all included studies is on average low.*

## It was shown that the summary effect of X versus S obtained from NMA is more likely to be statistically and clinically conclusive than the summary effect from a meta-analysis, because of indirect evidence derived via comparisons from other interventions. Do you think that NMA should be considered as the preferred evidence synthesis method instead of pairwise meta-analysis? [please say what **you think should be done**]

- Yes, NMA should always be preferred
- No, NMA should be considered only when we aim to synthesize evidence about more than two treatments
- It should be considered only if there is no direct evidence about X versus S
- It should be considered only if there are very few direct X versus S studies
- It should not be considered because the assumption of consistency is unlikely to hold in practice
- I don’t know
- Other (specify):

## Consider that NMA suggests that X is better than S while the pairwise meta-analysis suggests that more research is needed (as in the figure). Both analyses include studies with a low risk of bias, low risk of publication bias and the assumption of consistency is deemed reasonable (although the case of inconsistency can never be excluded). What do you conclude?

- I trust more the result from pairwise meta-analysis and I conclude that there is not enough evidence

0

-.4

-.2

.2

.4

Line of no effect

**meta-analysis**

**network meta-analysis**

Summary of standardized mean difference

*X is better*

*S is better*

- I trust the result from NMA and I conclude that the evidence favors intervention X for that particular outcome
- I don’t know what to conclude

Part II

***Part II has 9 questions.***

***IMPORTANT INSTRUCTIONS***

- *In the following questions the term ‘clinical trials’ we mean* ***randomized, post-marketing (e.g. phase IV) controlled clinical trials.***
- *We ask you to answer some of the questions under your capacity as a citizen who funds research through his taxation (e.g. EU funded clinical trials or other research conducted with national funds). We don’t ask you to pretend to be the average citizen, but somebody with your scientific skills, understanding and experience. Such questions start with* ***“As a citizen supporting publicly funded…”***

# Designing a future study

## Consider an important health condition for which we would like to know the optimal pharmacological treatment approach. There are some pairwise meta-analyses that compare pairs of drugs and although there are some indications that some drugs might be preferable to standard care, it is unclear which drugs(s) should be recommended. As a citizen supporting publicly funded research how would you rank (from 1 being top-priority to 5 being least priority) the following proposals tackling the treatments for that condition? Consider also the cost for each research proposal (presented in parenthesis in arbitrary units).

- A well-powered 3-arm randomized trial comparing the three most promising interventions (none of which is standard care) **(100)**
- A well-powered 3-arm randomized trial comparing the two most promising interventions and standard treatment **(90)**
- A well-powered 2-arm randomized trial comparing a newly launched treatment and standard treatment **(70)**
- A large registry involving many countries **(40)**
- A network meta-analysis comparing all available treatments using existing studies **(10)**

## According to your experience with clinical trials, how often does the design of a new post-marketing randomized trial considers the results of previous meta-analyses?

Never Always

______________________________________

## According to your experience with clinical trials, results from relevant meta-analyses are considered to:

- Define the alternative effect size in power calculations
- Decide about the intervention in the comparator arm
- Define other parameters involved in sample size calculations (e.g. standard deviation, baseline risk etc.)
- Other (specify)

## Which of the following statements best describes your beliefs about the role of meta-analysis in the likelihood to obtain public funding for a new clinical trial?

- The use of a meta-analysis results (in any way) to design the new trial increases the chances of obtaining public funding
- The funding application shall include some discussion about relevant existing meta-analyses but there is no need to use meta-analysis results to design the new trial
- Considering meta-analyses to design the new trial or discussion about existing relevant meta-analyses in the application would not materially affect the chances to obtain public funding.

*Typically, sample size calculations for a new trial are made assuming that the trial is a stand-alone experiment. Instead, we can calculate the sample size needed so that the trial adds enough power to an existing meta-analysis. Then, the required sample size for this new trial would be lower than what is traditionally calculated. The new trial might not be ‘statistically significant’ on its own, but the updated meta-analysis (the meta-analysis that includes the new study) would be powered enough to detect treatment effects, if present.*

## Where you aware of this approach?

- Yes
- No

## Would you be willing to consider such a design next time you plan a trial?

- Yes
- Possibly, if………..
- No, because…….

## What do you think is the biggest barrier towards adopting this new approach in designing trials? (choose only one option)

- Lack of training
- Changing the paradigm of funders and researchers
- Lack of good-quality meta-analyses
- Other (specify)

## As a citizen supporting publicly funded research would you think that priority should be given to such a trial design compared to conventional sample size calculations in order to minimize the number of patients we experiment with?

- Yes
- No
- I don’t know

## When designing a new trial aiming to evaluate the performance of a new intervention recently marketed, what best describes the practices applied when you choose the comparator arm. Assume that other, several alternative treatment options exist in the market.

- Most often the active comparator is the standard of care in the country the trial is going to take place
- Most often the active comparator is defined after a systematic review to identify the most efficacious and safe existing intervention
- Most often there is no active comparator arm - only no treatment, placebo or waiting list- although active alternatives do exist

***THANK you very much for your co-operation!***
