# Supplementary material for: Synthesizing existing evidence to design future trials: survey of methodologists from European institutions
Source: Trials. 2019 Jun 7;20:334. doi: 10.1186/s13063-019-3449-6 (PMC6555919; doi:10.1186/s13063-019-3449-6)
Supplement: Supplementary file 3 — Full results. (DOCX 45 kb) [file 13063_2019_3449_MOESM3_ESM.docx]

Additional file 3

# Appendix Tables

Appendix Table 1 Characteristics of survey participants. Questions have been simplified for presentation purposes. The full text and questions the participants were asked are available in Additional file 2.

| ***Question*** | Possible answers | Responses (%) |
| --- | --- | --- |
| ***Population characteristics (total participants: 76)*** | | |
| Have you been involved in any of the following: (more than one choice allowed) | Decide about funding clinical research (including reviewing grant applications)  Producing guidelines for clinical practice  Performing and/or evaluating systematic reviews and meta-analysis  Producing methodological guidance for systematic reviews and meta-analysis  Designing clinical trials  Conduct/support clinical trials  Consulting for clinical trials  Missing (skipped question) | 36 (47%)  33 (43%)  53 (70%)  28 (37%)  46 (61%)  44 (58%)  39 (51%)  1 (1%) |
| In how many trials have you been involved? | None  1-5  5-20  More than 20  Missing | 10 (13%)  19 (25%)  11 (15%)  33 (43%)  3 (4%) |
| In how many pairwise meta-analyses have you been involved? | None  1-5  5-20  More than 20  Missing | 14 (18%)  31 (41%)  16 (21%)  14 (18%)  1 (1%) |
| In how many network meta-analyses have you been involved? | None  1-5  5-20  More than 20  Missing | 40 (53%)  25 (33%)  6 (8%)  3 (4%)  2 (3%) |
| ***To subset of evidence synthesis experts participants (total participants, 68)*** | | |
| What is your level of experience with network meta-analysis? | I can perform network meta-analysis  I have been involved in systematic reviews with network meta-analysis but never done the statistical synthesis  I have read systematic reviews with network meta-analysis  I have heard about network meta-analysis but I don’t know much about it  I don’t know what a network meta-analysis is  Missing | 14 (21%)  13 (19%)  17 (25%)  8 (12%)  3 (4%)  13 (19%) |

Appendix Table 2 Opinions and practices of participants regarding the parts of the framework that answer the questions, “Does the existing evidence answer the research question?”. Questions have been simplified for display purposes. The full text and questions the participants were asked are available in Additional file 2.

| ***Question*** | Possible answers | Responses (%) |
| --- | --- | --- |
| ***Does the existing evidence answer the research question? (total participants: 68)*** | | |
| Assume that you want to repeat the meta-analysis every time that a new study is published until you have conclusive evidence. Do you **think** that adjustment for multiple testing is required when you form your conclusions? | Yes  No  I don’t know  Missing | 18 (27%)  22 (32%)  17 (25%)  11 (16%) |
| If your answer in the above question was yes, do you apply it in practice?  **(total participants: 18)** | Yes  No because I don’t know how  No because it is not common practice in my organization | 4 (22%)  4 (22%)  10 (56%) |
| How do you interpret evidence from multiple outcomes in forming your decision about the relative preference of two treatments (more than one choice allowed)? | We use some formal decision analysis approach to weigh the importance of beneficial and harmful outcomes (such as the benefit-harm trade-off method or multiple criteria decision analysis, etc.)  We use methods described in the ‘GRADE for recommendations’ approach to select a set of critical outcomes and then we summarize the results qualitatively  We involve stakeholders to decide which outcomes are more important than others  We don’t integrate results from different outcomes into a single conclusion  We summarize results qualitatively in an informal way  I haven’t been involved in the interpretation of results from a meta-analysis  Other (specify)  Missing | 11 (16%)  22 (32%)  25 (37%)  8 (12%)  14 (21%)  8 (12%)  8 (12%)  15 (22%) |

Appendix Table 3 Opinions and practices of participants regarding the part of the framework that answers the question, “How to use the existing evidence to plan future research?” Questions have been simplified for display purposes. The full text and questions the participants were asked are available in Additional file 2.

| ***How to use the existing evidence to plan future research? (total participants: 43)*** | | |
| --- | --- | --- |
| ***Question*** | Possible answers | Responses (%) |
| How is the comparator arm chosen in a new trial? | It is the standard of care in the country the trial is going to take place  It is defined after a systematic review to identify the most efficacious and safe existing intervention  Most often there is no active comparator arm - only no treatment, placebo or waiting list- although active alternatives do exist  Missing | 30 (70%)  3 (7%)  3 (7%)  7 (16%) |
| Which of the following statements  best describes your beliefs about the role of meta-analysis in the likelihood to obtain public funding for a new clinical trial? | The use of a meta-analysis (in any way) to design the new trial increases the chances of obtaining public funding  The funding application shall include some discussion about relevant existing meta-analyses but there is no need to use meta-analysis results to design the new trial  Considering meta-analyses to design the new trial or discussion about existing relevant meta-analyses in the application would not materially affect the chances to obtain public funding  Missing | 25 (58%)  9 (21%)  2 (5%)  7 (16%) |
| We can calculate the sample size needed so that the trial adds enough power to an existing meta-analysis. The new trial might not be statistically significant on its own, but the updated meta-analysis (the meta-analysis that includes the new study) would be powered enough to detect treatment effects, if present.  Are you aware of this approach? | Yes  No  Missing | 26 (60%)  11 (26%)  6 (14%) |
| Would you be willing to consider such a design next time you plan a trial? | Yes  Possibly  No  Missing | 10 (23%)  18 (42%)  9 (21%)  6 (14%) |
| As a citizen supporting publicly funded research would you think that priority should be given to such a trial design compared to conventional sample size calculations in order to minimize the number of patients we experiment with? | Yes  No  Missing | 21 (49%)  16 (37%)  6 (14%) |

Appendix Table 4 Rating frequencies and percentages of several research proposals, as resulted from asking the question “As a citizen supporting publicly funded research how would you rank (from 1 being the top priority to 5 being the least) the following proposals tackling the treatments for an important health condition? Consider also the cost for each research proposal (presented in parenthesis in arbitrary units)” to 43 participants experienced in clinical trial design.

| **Rating** | **1** | **2** | **3** | **4** | **5** | **Response Count** |
| --- | --- | --- | --- | --- | --- | --- |
| A well-powered 3-arm randomized trial comparing the three most promising interventions (none of which is standard care) (100) | 0 (0%) | 7 (16%) | 7 (16%) | 13 (30%) | 12 (28%) | 39 |
| A well-powered 3-arm randomized trial comparing the two most promising interventions and standard treatment (90) | 17 (40%) | 16 (37%) | 6 (14%) | 2 (5%) | 0 (0%) | 41 |
| A well-powered 2-arm randomized trial comparing a newly launched treatment and standard treatment (70) | 3 (7%) | 9 (21%) | 13 (30%) | 11 (26%) | 4 (9%) | 40 |
| A large registry involving many countries (40) | 1 (2%) | 4 (9%) | 5 (12%) | 9 (21%) | 20 (47%) | 39 |
| A network meta-analysis comparing all available treatments using existing studies (10) | 20 (47%) | 4 (9%) | 9 (21%) | 4 (9%) | 3 (7%) | 40 |

Appendix Table 5 Association between evidence synthesis experts (68 participants) and experience with network meta-analysis, and their opinions on whether network meta-analysis should be considered the preferred evidence synthesis method.

|  | | **Network meta-analysis should be preferred** | |  |
| --- | --- | --- | --- | --- |
|  |  | **No** | **Yes** | **Total** |
| **Can perform network meta-analysis or have been involved in systematic reviews with network meta-analysis** | **No** | 37 (54%) | 4 (6%) | 41 (60%) |
|  | **Yes** | 16 (24%) | 11 (16%) | 27 (40%) |
|  | **Total** | 53 (78%) | 15 (22%) | 68 (100%) |

Answers to the question “What is your level of experience with network meta-analysis?” have been grouped in two categories; the first includes “I can perform network meta-analysis” and “I have been involved in systematic reviews with network meta-analysis but never done the statistical analysis” and the second includes the remaining answers. Answers to the question “Should network meta-analysis be considered the preferred evidence synthesis method?” have been grouped in two categories; the first includes “Yes, network meta-analysis should always be preferred” and the second includes the remaining answers. Pearson’s Chi-squared test P-value: 0.003

Appendix Table 6 Association between clinical trial design experts (43 participants, 6 missing values) experience on clinical trials and their willingness to consider calculating sample size based on an existing meta-analysis next time they plan a trial.

|  | | **Would consider sample size calculations based on meta-analysis** | |  |
| --- | --- | --- | --- | --- |
|  |  | **No** | **Yes / possibly** | **Total** |
| **Number of clinical trials involved** | **More than five** | 8 (22%) | 19 (51%) | 27 (73%) |
|  | **None to five** | 1 (3%) | 9 (24%) | 10 (27%) |
|  | **Total** | 9 (24%) | 28 (76%) | 37 (100%) |

Answers to the question “In how many trials have you been involved?” have been grouped in two categories; “involved in more than five clinical trials” and “involved in none to five clinical trials.” Answers to the question “Would you be willing to consider such a design next time you plan a trial?” have been grouped in two categories; the first includes “No” and the second includes “Yes” and “Possibly.” Fisher’s exact test P-value: 0.393.

Appendix Table 7 Association between clinical trial design experts (43 participants, 6 missing values) experience on evidence synthesis and their willingness to consider calculating sample size based on an existing meta-analysis next time they plan a trial.

|  |  | **Would consider sample size calculations based on meta-analysis** | |  |
| --- | --- | --- | --- | --- |
|  |  | **No** | **Yes/possibly** | **Total** |
| **Number of pairwise or network meta-analyses involved** | **More than five** | 4 (11%) | 15 (41%) | 19 (51%) |
|  | **None to five** | 5 (14%) | 13 (35%) | 18 (49%) |
|  | **Total** | 9 (24%) | 28 (76%) | 37 (100%) |

Answers to the question “In how many meta-analyses have you been involved?” and to the question “In how many network meta-analyses have you been involved?” have been combined to form two categories; “involved in either more than five pairwise meta-analyses or more than five network meta-analyses” and “involved in none to five pairwise and none to five network meta-analyses.” Answers to the question “Would you be willing to consider such a design next time you plan a trial?” have been grouped in two categories; the first includes “No” and the second includes “Yes” and “Possibly.” Fisher’s exact test P-value: 0.714.
